# Effect of combined siRNA of HCV E2 gene and HCV receptors against HCV

**DOI:** 10.1186/1743-422X-8-295

**Published:** 2011-06-10

**Authors:** Shah Jahan, Saba Khaliq, Baila Samreen, Bushra Ijaz, Mahwish Khan, Waqar Ahmad, Usman Alli A Ashfaq, Sajida Hassan

**Affiliations:** 1 Applied and Functional Genomics Lab, Centre of Excellence in Molecular Biology, University of the Punjab, 87 West Canal Bank Road Thokar Niaz Baig, Lahore, Pakistan; 2 Division of Molecular Medicine, Centre of Excellence in Molecular Biology, University of the Punjab, Lahore, Pakistan

**Keywords:** HCV, siRNA, HCV receptors, HCV envelope genes and viral titer

## Abstract

**Background/Aim:**

Hepatitis C virus (HCV) is a major threat as almost 3% of the world's population (350 million individual) and 10% of the Pakistani population is chronically infected with this virus. RNA interference (RNAi), a sequence-specific degradation process of RNA, has potential to be used as a powerful alternative molecular therapeutic approach in spite of the current therapy of interferon-α and ribavirin against HCV which has limited efficiency. HCV structural gene E2 is mainly involved in viral cell entry via attachment with the host cell surface receptors i.e., CD81 tetraspanin, low density lipoprotein receptor (LDLR), scavenger receptor class B type 1 (SR-B1), and Claudin1 (CLDN1). Considering the importance of HCV E2 gene and cellular receptors in virus infection and silencing effects of RNAi, the current study was designed to target the cellular and viral factors as new therapeutic options in limiting HCV infection.

**Results:**

In this study the potential of siRNAs to inhibit HCV-3a replication in serum-infected Huh-7 cells was investigated by combined treatment of siRNAs against the HCV E2 gene and HCV cellular receptors (CD81 and LDLR), which resulted in a significant decrease in HCV viral copy number.

**Conclusion:**

From the current study it is concluded that the combined RNAi-mediated silencing of HCV E2 and HCV receptors is important for the development of effective siRNA-based therapeutic option against HCV-3a.

## Introduction

HCV infection is a major health problem with nearly 10% chronically infected population in Pakistan and 350 million people worldwide [1,2]. About 75% of patients achieve no therapeutic benefit from the present combination therapy with pegylated interferon α (PEG-IFN-α) and ribavirin mainly depending upon HCV genotype, whereas in 40-60% patients chronic infection is mainly associated with liver cirrhosis and steatosis leading to hepatocellular carcinoma (HCC) [3-5]. In Pakistan the major HCV genotype is 3a followed by 3b and 1a, with a strong correlation between chronic HCV infection and HCC in Pakistan associated with genotype 3a [6,7]. There is a desperate need to develop more efficient and better therapeutic alternative for treatment of HCV infections.

Due to the absence of suitable animal model and competent in vitro cell culture system the mechanism of HCV cell entry was unrevealed after a long time. Recently, different groups have studied HCV replication in serum infected liver cell lines which mimics the naturally occurring HCV virions biology and kinetics of HCV infection in humans liver cells [8-11]. HCV envelop glycoproteins E1 and E2 are involved in HCV entry, fusion and defense against neutralization by envelop-specific host antibodies [12-18]. E2 glycoprotein works as a key component in interaction between the virus and its major cellular receptors i.e., CD81, SR-BI and CLDN1 [15-17]. CD81 is a main HCV cell surface receptor, whereas additional role is played by the scavenger receptor class B type I (SRBI) and the low-density-lipoproteins receptor (LDLR) [19-22]. LDLR is potentially involved in the uptake of lipoprotein-associated HCV into hepatocytes as serum fraction composed of HCV with LDL, or very low-density lipoprotein (VLDL), which are involved in binding to the LDL receptor as a possible mechanism of HCV cell entry [23-25]. Hence, HCV envelope glycoprotein and cellular receptors is good target for the development of antiviral molecules that could block HCV entry.

Being a RNA virus HCV is highly susceptible to RNA interference (RNAi) induced by small interfering RNA (siRNA), which is a sequence specific gene silencing mechanism [26-28]. siRNAs can be used as a potential therapeutic agent against HCV because HCV replication takes place in the cytoplasm of liver cells without integration into the host genome. siRNA directed against HCV genotype 1a and 1b has been shown to effectively block the replication of viral replicons in Huh-7-derived cell lines [29-35]. In our previous study, the development of siRNA targeting envelope proteins of the local HCV-3a genotype showed that these genes are crucial for viral entry providing better choice for developing a rational antiviral strategy against HCV [36]. Several investigators have reported the inhibition of HCV RNA by targeting structural and non structural genes of HCV and cellular genes by using siRNAs in combination [33,36-38]. In this report, we investigated the effect of siRNA induced silencing of receptor genes and HCV E2 on viral load of HCV followed by a combined effect which showed a significantly decreased viral RNA.

## Results

**Figure 1:**
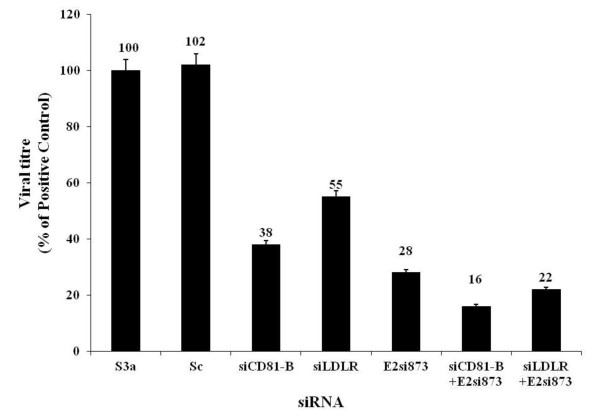
**Graphical representation of combine effect of siRNA against HCV E2 gene and HCV receptors gene CD-81, LDLR on viral titer in serum infected Huh-7 cells**. For Viral titer in Huh-7 cells treated with scrambled siRNAs (Sc) and CD-81 siRNA (siCD81-B), LDL siRNA (siLDLR), E2 siRNA (E2si873) either alone or in combinations and incubated for 6 hrs before adding HCV-3a sera (Ser 3a). HCV RNA levels were quantified by Real Time PCR. Three independent experiments with triplicate determinations were performed. Error bars indicate, mean S.D *p < 0.01 verses Ser3a.

Cellular genes CD81 and LDLR are functionally involved in HCV entry. Our previous results also show that sequence specific siRNAs against each receptor significantly inhibit the expression of their respective genes of receptors CD81, LDLR, SRBI and CLDN1 (data submitted for publication). Keeping all these in view, we used *in-vitro *transcribed siRNA against HCV E2 gene and cellular receptors CD81and LDLR and observed the effect of silencing of these receptors on viral titer. Previously, we have successfully inhibited HCV by E2 siRNAs [36], from that study we selected the best one for this study. The effect on viral titer was analyzed by silencing each receptor and E2 gene individually and then in combination of siRNAs against two receptors and E2 gene simultaneously. To evaluate the role of HCV E2 gene and HCV receptors in HCV infection and HCV pathogenesis, Huh-7 cells were infected with HCV-3a serum with or without siRNAs against HCV receptors CD81, LDLR and HCV E2-3a gene for 48hrs and viral loads were quantified by Real Time PCR. Results showed a decrease of 62%, 45%, with HCV receptor siRNA CD-81, LDL, 60%, and 72% with HCV E2 siRNA, (E2si873), respectively in viral load. A significant suppression of HCV RNA (84% and 78%) was observed with the combination of both siRNAs against HCV E2 gene, E2 siRNA (E2si873) and HCV receptor genes CD81(siCD81-B), LDLR (siLDLR) (Figure 1).

**Figure 2:**
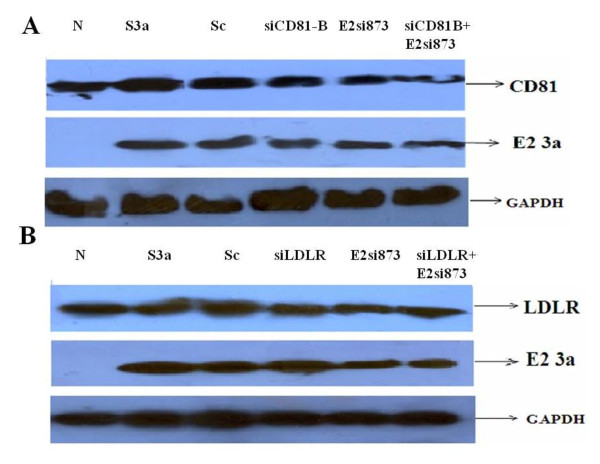
**Protein expression analysis of HCV E2 gene and HCV receptors genes by using siRNA alone and in combination against HCV gene E2 and HCV receptor genes CD81, LDLR**. Protein isolated from Huh-7 cells treated with single and combination of siRNA against HCV E2 gene and receptor CD81 and LDLR genes and incubated for 6 hrs before adding HCV-3a sera (Ser 3a) for 48 hrs. Protein levels were quantified by western blot analysis using specific antibodies of CD81, LDLR, E2 and GAPDH. **A) **Silencing of CD81 gene and HCV 3a E2 gene alone and in combination using specific antibodies showing reduction at protein expression level. **B) **Silencing of LDLR gene and HCV 3a E2 gene alone and in combination, using specific antibodies showing reduction at protein expression level. Protein levels for GAPDH gene are also shown as internal control and scramble siRNA (Sc) as siRNA control.

Furthermore, the effect on protein expression inhibition of HCV structural gene E2, receptor genes CD81 and LDLR on the expression of viral protein were determine by western blot analysis using specific antibodies. Huh-7 cell lysates infected with HCV serum of genotype 3a with or without siRNAs (100nM each) against HCV receptors CD81, LDLR and HCV E2-3a gene for 48hrs were separated through SDS PAGE and treated with specific antibodies of each gene. Results indicate the significant inhibition of protein expression of CD81 and E2 3a, when combination of siRNA (E2si873 +siCD81-B) were used as compare to individual siRNA against CD81 and HCV E2 (Figure 2A). These results show the reduce total cellular viral protein expression due to the low expression of HCV envelop protein as well as CD81 receptor protein simultaneously. Similarly, western blotting results indicate the significant inhibition of expression of LDLR and E2 3a, when combination of siRNA (E2si873 +siLDLR) were used as compare to individual siRNA against LDLR and HCV E2 (Figure 2B), which also show the reduce total cellular viral protein expression due to the low expression of HCV envelop protein as well as LDLR receptor protein simultaneously.

## Discussion

HCV entry into hepatocytes, a multistep process mediated by HCV envelop glycoprotein E1 and E2 and several cell surface receptors, is first step of virus life cycle that results in productive viral infection, providing major targets for immunopreventive and therapeutic strategies [39-41]. The cell surface receptors mainly include tetraspanin protein CD81, SR-BI, LDLR and CLDN1 a tight junction protein [17,20-22,42]. HCV envelop protein E2 posses' glycosylation sites which interact directly with these cell surface receptors. As in our previous study, here too we utilized serum infected Huh-7 cell culture model to evaluate the effect of siRNA separately and in combination against HCV structural gene and receptor genes on viral entry by quantifying the viral titer in siRNA-treated and non-treated serum-infected Huh-7 cells.

RNAi is an exciting new therapeutic technology proposed to be used in treatment of viral diseases. HCV is an attractive target for RNAi therapy as its genome is a (+) sense single stranded RNA that functions as both the viral messenger RNA and template for RNA replication via negative strand intermediate. Previously it has been reported that cellular genes functionally involved in HCV entry like CD81, LDLR, SR-BI and CLDN1 also serve as potential targets for RNAi. Several reports showed potent RNAi against HCV genes and cellular receptors activity to reduce the HCV infection in which expression of HCV was distinctly inhibit HCV serum infection (30%-90%) [11,36,42-45]. In our current project, we utilized siRNAs to silence the expression of HCV cellular receptors and E2 gene to block the HCV entry in serum derived HCV infected Huh-7 cell culture model and analyze its effect on viral load. HCV infection pathway employs enhancement in expression of cell surface receptors that may facilitates to increase viral load. In account of these, we knock down the expression of host cell surface HCV receptors by using siRNA to block HCV entry, against each receptor gene separately and in combination of siRNA against two receptors gene in Huh-7 cells which were further infected with HCV serum of genotype 3a and observed the viral titer by detection of 5'UTR of viral copies by Real Time PCR in cells from 3^rd ^day post infection. Our results indicate significant decrease in HCV viral load by 67% and 58% due to the silencing of HCV receptor CD81 (33 fold) and LDLR (42 fold) respectively when compared to control (S3a), (Figure 1). Since, LDLR is important for HCV-E1-pseudotype infectivity, whereas CD81 determines the infectivity of HCV-E2-pseudotype virus, the silencing effect of siRNAs against selected HCV infection host cellular proteins has been evaluated to reduce viral titer significantly. Thus use of combinations of siRNAs against both the virus and host genes involve in HCV infection are likely to be a potent approach in the treatment of chronic hepatitis C due to their additive HCV RNA inhibition effects. Moreover, different studies exhibit the feasibility of targeting host cellular factors involved in infection, as they are not prone to mutations, as potential targets for siRNA therapy. Henry and colleagues [46] targeted the IRES, NS5B, and host cell receptor CD81 by triple shRNA expression vector which concurrently reduced the HCV replication, CD81 expression, and E2 binding. Targeting multiple sites of the HCV genome and host factors involved in HCV infection are a realistic and valid approach aimed at preventing the virus from developing resistance.

In correspondence to the latest reports, we also investigated the down regulation of viral titer by silencing the expressions of HCV envelop gene alone using HCV E2 specific siRNA (E2si873) and in combination with the silencing of CD81 or LDLR gene expression using siCD81-B, siLDLR in Huh-7 cells which were further infected HCV 3a serum. Our findings showed a significant decrease in HCV viral titer up to 67% and 58%, with siCD81-B, siLDLR and 72% with HCV E2 siRNA (E2si873), respectively in the Huh-7 cells. A significant suppression of HCV RNA (84% and 78%) was observed with the combination of both siRNAs against HCV E2 gene, E2 siRNA (E2si873), and CD81, LDLR siRNAs (Figure 1 ). Likewise, cell lysates from HCV serum infected Huh-7 cells were examined by western blot analysis using CD81, LDLR and HCV-E2 specific antibodies. Our results showed considerable decreased in the protein levels of CD81, LDLR and HCV-E2 in transiently transfected siCD81-B, siLDLR and E2si873 siRNAs, whereas combination of siCD81-B + E2si873 and E2si873 + siLDLR resulted in a more significant decrease of CD81, LDLR protein expression that ultimately reduced the protein level of HCV-E2 which depicts the reduced entry of HCV, hence lessen the HCV infection (Figure 2).

In summary, our data showed that CD81 and LDL specific siRNAs not only reduced their gene expression respectively but also reduced viral titer in siRNA treated cells confirming their role in HCV infection; combination of these siRNA (siCD81-B, siLDLR) with E2 effective siRNA (E2si873) showed dramatic reduction of HCV entry. Use of siRNA to inhibit the HCV E2 protein or HCV receptor protein expression alone or in combination could be helpful in reduction of HCV entry. In addition, we propose the use of combination siRNAs against HCV gene with host genes which could inhibit HCV entry better than separately used siRNA.

## Materials and Methods

### Source of samples

The local HCV-3a patient's serum samples used in this investigation were obtained from the CAMB (Center for Applied Molecular Biology) diagnostic laboratory, Lahore, Pakistan after quantification and genotype determination. Serum samples were stored at -80°C prior to RNA extraction for cloning and viral inoculation experiments. Patient's written consent and approval for this study was obtained from institutional ethics committee.

### Designing and synthesis of siRNA

Designing and synthesis of siRNA were done as we have described earlier [36]. siRNA oligonucleotides were designed to express RNAi mechanism against host HCV receptors (LDLR and CD81) and E2 region of HCV-3a genome using the Ambion's siRNA design tool http://www.ambion.com/techlib/misc/siRNA_finder.html after sequencing of local HCV-3a patient's serum samples (Table 1). The designed siRNAs (cellular genes HCV receptors, HCV-3a E2 and control Scrambled) were synthesized using Silencer siRNA construction kit according to the manufacturer's instruction (Ambion, USA).

**Table 1:** 

Name	Sequences
Scramble-antisense	AACCTGCATACGCGACTCGACCCTGTCTC
Scramble-sense	AAGTCGAGTCGCGTATGCAGGCCTGTCTC
CD81-B antisense	AAGATGCCTACATAGAAGGTGCCTGTCTC
CD81-B sense	AACACCTTCTATGTAGGCATCCCTGTCTC
LDL antisense	AAATGCATCTCCTACAAGTGGCCTGTCTC
LDL sense	AACCACTTGTAGGAGATGCATCCTGTCTC
E2si873-antisense	AACAACTGAGCTTGCCATACTCCTGTCTC
E2si873-sense	AAAGTATGGCAAGCTCAGTTGCCTGTCTC

### Viral inoculation and co-transfection with siRNA

Huh-7 cell line was kindly provided by Dr. Zafar Nawaz (University of Miami, USA) and maintained in Dulbecco's modified eagle medium (DMEM) supplemented with 100 μg/ml penicillin; streptomycin and 10% fetal bovine serum referred as complete medium (Sigma Aldrich, USA) at 37°C with 5% CO_2_. The medium was renewed every 3 day and passaged every 4-5 days. Huh-7 cell line was used to establish the in vitro replication of HCV genotype 3a. A similar protocol was used for viral inoculation as described by earlier [36,45]. For these experiments high viral titer > 1 × 10^8 ^IU/ml containing serum from HCV-3a patient was used as principle inoculum. Huh-7 cells were maintained in 6-well culture plates to semi-confluence, washed twice with serum-free medium then inoculated with 500 μl (5 × 10^7^IU/well) viral load of HCV-3a sera and 500 μl serum free media. Cells were maintained overnight at 37°C in 5% CO_2_. Next day, the adherent cells were washed three times with 1 × PBS, complete medium was added and incubation was continued for 48hrs. Cells were harvested and assessed for the presence of viral RNA quantitatively by Real Time PCR. To analyze the effect of siRNA on HCV infection, serum infected Huh-7 cells were seeded after three days of infection in 24-well plates and grown to 80% confluence with 2ml medium. The cells were transfected with or without 40 μM/well cellular receptors CD81, LDL-R and E2 siRNAs alone or in combination using Lipofectamine™ 2000 (Invitrogen Life technologies, CA) according to the manufacturer's protocol.

### Viral load quantification

Cells were harvested for viral load determination using Gentra RNA isolation kit (Gentra System Pennsylvania, USA) according to the manufacturer's instructions. For viral quantification Sacace HCV quantitative analysis kit (Sacace Biotechnologies Caserta, Italy) was used. Briefly, 10 μl of extracted viral RNA was mixed with an internal control provided by Sacace HCV Real TM Quant kit and subjected to viral quantification using Real Time PCR SmartCycler II system (Cepheid Sunnyvale, USA).

### Total RNA isolation and gene expression analysis

Total RNA from HCV serum infected and non-infected cells was isolated using TRIzol reagent (Invitrogen life technologies, CA), 24 hrs and 48hrs post-transfection. To analyze the effect of siRNA on envelope gene expression, cDNA was synthesized with 1 μg of total RNA using Superscript III cDNA synthesis kit (Invitrogen life technologies, CA) and semi-quantitative RT-PCR was done using primers of HCV receptors, E2 genes and GAPDH as control. Quantitative Real Time PCR was carried out using Real Time ABI 7500 system (Applied Biosystems Inc, USA) with SYBR Green mix (Fermentas International Inc, Canada) as we have described earlier [36]. The relative gene expression analysis was carried out by the SDS 3.1 software (Applied Biosystems Inc, USA). Each individual experiment was performed in triplicate.

### Western blotting

To determine the effect of siRNAs on protein expression levels HCV receptors CD81 and LDL-R and E2, in HCV serum infected cells, cells were lysed using ProteoJET mammalian cell lysis reagent (Fermentas, Canada). Equal amounts of total proteins were subjected to electrophoresis on 12% SDS-PAGE and electrophoretically transferred to a nitrocellulose membrane according to the manufacturer's protocol (Bio-Rad, CA). After blocking non-specific binding sites with 5% skimmed milk, blots were incubated with primary monoclonal antibodies specific to HCV cellular receptors like CD81 and LDL-R, HCV E2 and GAPDH genes (Santa Cruz Biotechnology Inc, USA) and secondary Horseradish peroxidase-conjugated anti-goat anti-mouse antibody (Sigma Aldrich, USA). The protein expressions were evaluated using chemiluminescence's detection kit (Sigma Aldrich, USA).

### Statistical analysis

All statistical analysis was done using SPSS software (version 16.0, SPSS Inc). Data are presented as mean ± SD. Numerical data were analyzed using student's t-test and ANOVA. P value < 0.05 was considered statistically significant.

## List of abbreviations

E1, E2: Envelop proteins 1, 2, HCC: Hepatocellular carcinoma, HCV: Hepatitis C virus, CD81: Cluster of differentiation 81, SR-BI: Scavenger Receptor Class B Type I, LDL-R: Low-Density Lipoprotein Receptor, CLDN 1: Claudin 1, PEG-INF-α: pegylated interferon alpha, RNAi: RNA interference, siRNAs: small interfering RNAs.

## Authors' contributions

SJ, SK and BS contributed equally to this work, conceive the idea and performed all the lab work. MH and UAA helped SJ and SK in lab work and literature review. BI and WA helped SJ and SK in data analysis. SK and SJ critically reviewed and finalized the manuscript. SH provided all facilitates to complete this work. All authors read and approved the final manuscript.

## Authors' information

Shah Jahan (PhD Molecular Biology), Saba Khaliq (PhD Molecular Biology), Bailla Samreen (M.Phil Molecular Biology), Mahwish Khan (M.Phil Molecular Biolohgy) and Usman Ali Ashfaq (PhD Molecular Biology) are research scholars at CEMB. Bushra Ijaz (M.Phil Molecular Biology) and Waqar Ahmad (M.Phil Chemistry) are research officers at CEMB whereas Sajida Hassan (PhD Molecular Biology) principle investigator at CEMB, University of the Punjab, Lahore.

## Competing interests

The authors declare that they have no competing interests.
